# Low Levels of Blood Lipids Are Associated with Etiology and Lethal Outcome in Acute Liver Failure

**DOI:** 10.1371/journal.pone.0102351

**Published:** 2014-07-15

**Authors:** Paul Manka, Verena Olliges, Lars P. Bechmann, Martin Schlattjan, Christoph Jochum, Jürgen W. Treckmann, Fuat H. Saner, Guido Gerken, Wing-Kin Syn, Ali Canbay

**Affiliations:** 1 Department of Gastroenterology and Hepatology, University Hospital, University Duisburg-Essen, Essen, Germany; 2 Department of General, Visceral and Transplantation Surgery, University Hospital, University Duisburg-Essen, Essen, Germany; 3 Liver Regeneration and Repair, The Institute of Hepatology, Foundation for Liver Research, London, United Kingdom; 4 Department of Hepatology, Barts Health NHS Trust, London, United Kingdom; UMR Inserm U1052/CNRS 5286, France

## Abstract

**Background/Aims:**

Emerging data links different aspects of lipid metabolism to liver regeneration. In patients with acute liver failure (ALF), low levels of lipids may correlate with disease severity. Thus, we determined whether there is an etiology-specific link between lipid levels in patients suffering from ALF and aimed to investigate an effect of lipid levels on the prognosis of ALF.

**Methods:**

In this retrospective single center study, we reviewed 89 consecutive ALF patients, who met the criteria of the “Acute Liver Failure Study Group”. Patient characteristics, clinical data and laboratory parameters were individually analyzed at admission and correlated with the patients' outcome after a four week follow up. Possible endpoints were either discharge, or death or liver transplantation.

**Results:**

High-density lipoprotein (HDL), cholesterol and triglyceride levels were significantly lower in patients who died or required a liver transplant. HDL levels were significantly higher in patients with ALF caused by acetaminophen intoxication, compared to fulminant HBV infection or drug induced liver injury. HDL levels correlated with hepatic injury by ALT levels, and Albumin, and inversely correlated with the MELD score, INR, and bilirubin.

**Conclusion:**

In our cohort of patients with ALF, we could show that HDL and cholesterol are suppressed. In addition novel etiology specific patterns between acteminophen and non-acteminophen induced liver failure were detected for serum lipid components. Further studies are needed to address the role of cholesterol and lipid metabolism and the according pathways in different etiologies of ALF.

## Introduction

High density lipoprotein (HDL) is a major lipid component of the serum. HDL serves as transport molecule for cholesterol to various sites in the organism, among them the adrenal gland for steroidogenesis [1] but also to the liver as building block for cell membranes and as energy supply [2]. These two functions are especially important in times of tissue damage and repair or regeneration. HDL consists mainly of cholesterol and apolipoprotein A-I, which is produced and metabolized to a large extent in the liver [3].

In chronic liver diseases HDL is often found reduced [4], [5] and low levels are associated with an adverse disease course. For example low HDL concentrations are associated with poor outcome in sepsis [6]. This may be connected to the innate immune system, as HDL also binds lipopolysaccharides (LPS) of bacterial cell walls [7], [8]. Reduced liver function could thus indirectly aggravate systemic infections or inflammatory processes due to higher LPS concentrations.

Acute liver failure (ALF) represents a sudden loss of liver function, mostly due to death of large numbers of hepatocytes, which can be caused by a wide range of toxins, viral agents, autoimmune processes, and many more [9]. Since cell mass has to be restored during ALF, the regenerative capacity of the remaining liver cells, including the progenitor cell compartment, is a crucial factor for survival without a liver transplantation [10]. Resources of the remaining hepatocytes have to be distributed between normal liver function and proliferation. Thus, supply of energy and building blocks possibly contributes to the outcome of ALF. Since HDL serves both functions, it may be one important factor for outcome of ALF.

As previous studies have shown, low HDL is indeed associated with adverse outcome of ALF [11]. In the present study we aimed to confirm (i) reduced lipid components in ALF and (ii) that low lipid components, in particular HDL, are associated with poor outcome in ALF. Furthermore, we analyzed if (i) HDL has a predictive value for prognosis of ALF outcome and (ii) if HDL levels differ between various etiologies of ALF, as possible indicator for etiology specific disease course and outcome.

## Methods

### Patient information, data collections and ethical considerations

The study was carried out according to the Declaration of Helsinki and the guidelines of the International Conference for Harmonization for Good Clinical Practice. The study was approved by the local institutional review board (Ethik Kommission am Universitätsklinikum Essen). Due to the retrospective nature of the study, written informed consent could not be obtained from all patients. All data was de-identified prior to analysis.

In a retrospective single center study (11/2006–12/2010), we recruited 89 consecutive ALF patients (64% females/36% males), who met the criteria defined by the “Acute Liver Failure Study Group Germany” [9]. Briefly, ALF was diagnosed by the assessment of laboratory parameters (bilirubin, AST, ALT, AP, γ-GT), the international normalized ratio (INR), and neurological status. INR>1.5 and the presence of any degree of encephalopathy were defined as severe liver failure. Other causes of liver dysfunction were excluded, such as acute-on-chronic liver failure or pre-existing cirrhosis. All patients presented within four weeks of disease onset and had no pre-existing liver disease. Upon admission clinical data and lipid levels were collected. Outcome was defined as either spontaneous recovery (SR) or non-spontaneous recovery (NSR) at 4 weeks after admission.

### Laboratory parameters

Total cholesterol (TC), HDL-cholesterol and triglycerides (TG) were measured using an ADVIA Clinical Chemistry system (Siemens AG, healthcare sector; Munich, Germany). Individual values of clinical and standard laboratory data were assessed.

### Statistical analysis

Descriptive statistics are expressed as means +/− SD for normal distributed data otherwise median +/− range was used. All variables were tested for normal distribution using the Kolmogorov-Smirnov and the Shapiro-Wilk test. The student-t-test was used to compare continuous variables between groups. Otherwise, the Mann-Whitney-U-Test was used. Categorical data were tested using the Chi-square test. One-way ANOVA test followed by Bonferroni-Test was used for multiple comparisons.

## Results

### Demographic data

Mean age of the 89 patients [58 (65%) females and 31 (35%) males] was 38.8±15.2. Detailed laboratory parameters and patients' characteristics are listed in table 1. Sixty-one (67%) patients recovered spontaneously (SR), twenty-eight (33%) died or underwent LTx (NSR). Thirty-five patients (39%) had liver injury from drug-induced causes (acetaminophen in 15 cases, idiosyncratic drug induced liver injury (DILI) in 20 cases). Fifty-four patients had liver injury from non-drug causes (20 hepatitis B, 5 hepatitis A, 9 autoimmune, 6 miscellaneous and 14 indeterminate cases).

**Table 1:** Lipids in patients with acute liver failure.

Parameters (n)	Normal range	Total patient Mean +/− SD (SEM)	NSR Mean +/− SD (SEM)	SR Mean +/− SD (SEM)
Total cholesterol (88)	70–170 mg/dl	120.98+/−55.3 (5.9)	81.58+/−39.6 (8.085)	137.24+/−46.299 (7.231)
Triglycerides (63)	<150 mg/dl	143.32+/−91.843 (11.39)	130.75+/−80.367 (16.405)	150.68+/−98.42 (15.327)
LDL (87)	70–180 mg/dl	62.72+/−46.24 (4.901)	39.63+/−21.647 (4.419)	72.8+/−50.233 (7.845)
HDL (89)	>40 mg/dl	16.31+/−16.355 (1.734)	6.96+/−8.590 (1,753)	17.63+/−13.879 (2.168)

### Low lipid levels indicate lethal outcome or need for transplantation in ALF

**Figure 1:**
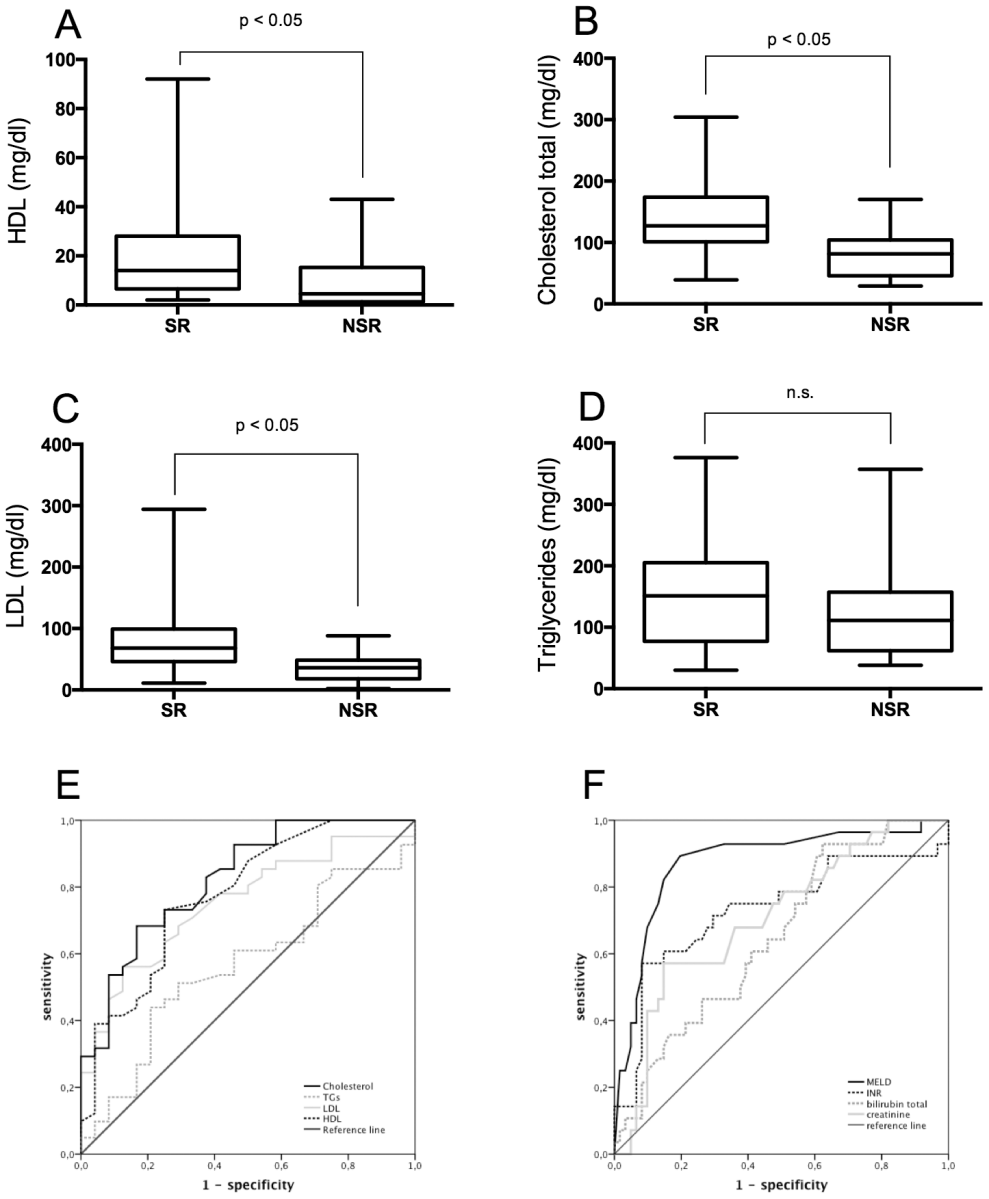
Lipid levels in acute liver failure. Lipid-levels were assessed during diagnostic procedures in ALF patients. Recordings are depicted for the first detected value within one week after admission (**A**) HDL; (**B**) total cholesterol; (**C**) LDL; (**D**) Triglycerides; All data are presented as median, *p vs. SR<0.05. To assess predictive properties of the analyzed parameters receiver operating characteristic (ROC) curves were calculated for lipid components (**E**), the MELD, and individual MELD components (**F**). The area under the receiver operating characteristic curve (AUROC) was 0.77 for HDL and 0.84 for total Cholesterol (**E**). A similar performance was reached for the MELD Score with an AUROC of 0.875 (**F**).

Lipid profiles were determined within one week from the time of hospital admission. Median TC and TG were within normal ranges (<200 mg/dl blood cholesterol considered as normal). Median HDL and LDL were below the normal ranges (table 1). SR patients exhibited a different lipid profile than the NSR group. HDL, LDL and TC were significantly lower in NSR compared to SR. TG levels were similar in SR and NSR (fig. 1 A–D).

Receiver operating characteristic (ROC) curves were calculated for the determined lipid parameters, the MELD, and individual MELD components. The area under the receiver operating characteristic curve (AUROC) was 0.77 for HDL and 0.84 for total Cholesterol (fig. 1E) A threshold level of 6.5 mmol/l for HDL (sensitivity: 73.2%, specificity: 75%, AC: 0.73) and 103.5 mmol/l for total cholesterol (sensitivity: 73%, specificity: 70%, AC: 0.71) resulted in optimal discrimination between SR and NSR. The predictive value proved to be similar to the calculated MELD Score with an AUROC of 0.875. (fig. 1F). Parameters of infection (leukocyte count and CRP) failed to show any association with outcome (data not shown).

### Serum HDL correlates with transaminase levels

**Figure 2:**
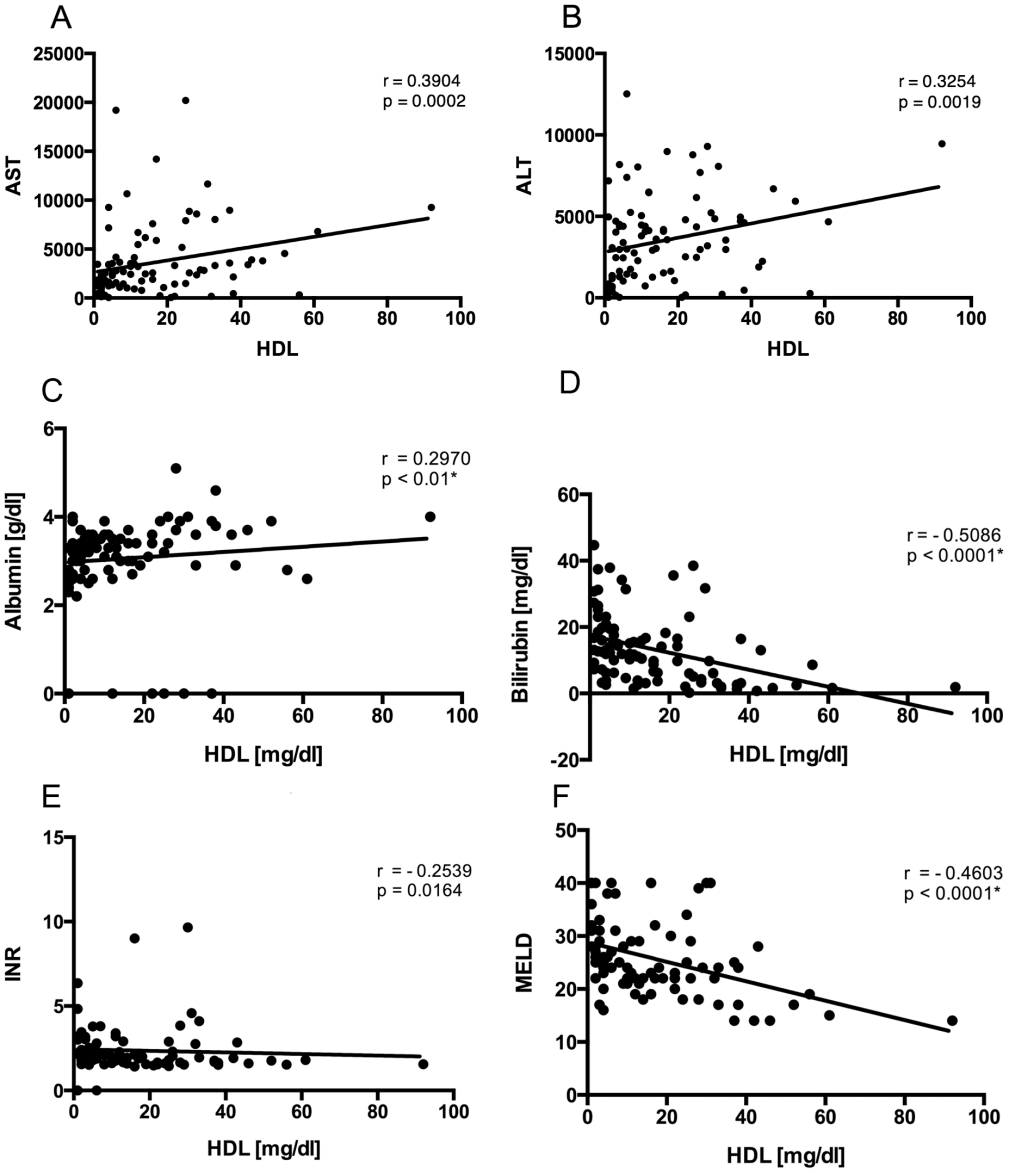
Comparison between HDL and parameters of cell-damage and cell-death. We found a positive correlation for HDL and AST (A), ALT (B), and Albumin (C) as parameters of liver function. In contrast, bilirubin (D), international normalized ratio (INR, E), and the MELD score (F) as indicators for liver damage were inversely correlated to HDL.

Elevation of transaminase levels suggests severe liver damage. In our cohort, we found a positive correlation for HDL and AST as well as ALT (fig. 2A, B). HDL also correlated to serum Albumin as functional liver parameter (fig. 2C). Negative correlations were found for HDL and bilirubin (fig. 2D), INR (fig. 2E), and the MELD score (fig. 2F).

### HDL levels differ between different etiologies

**Figure 3:**
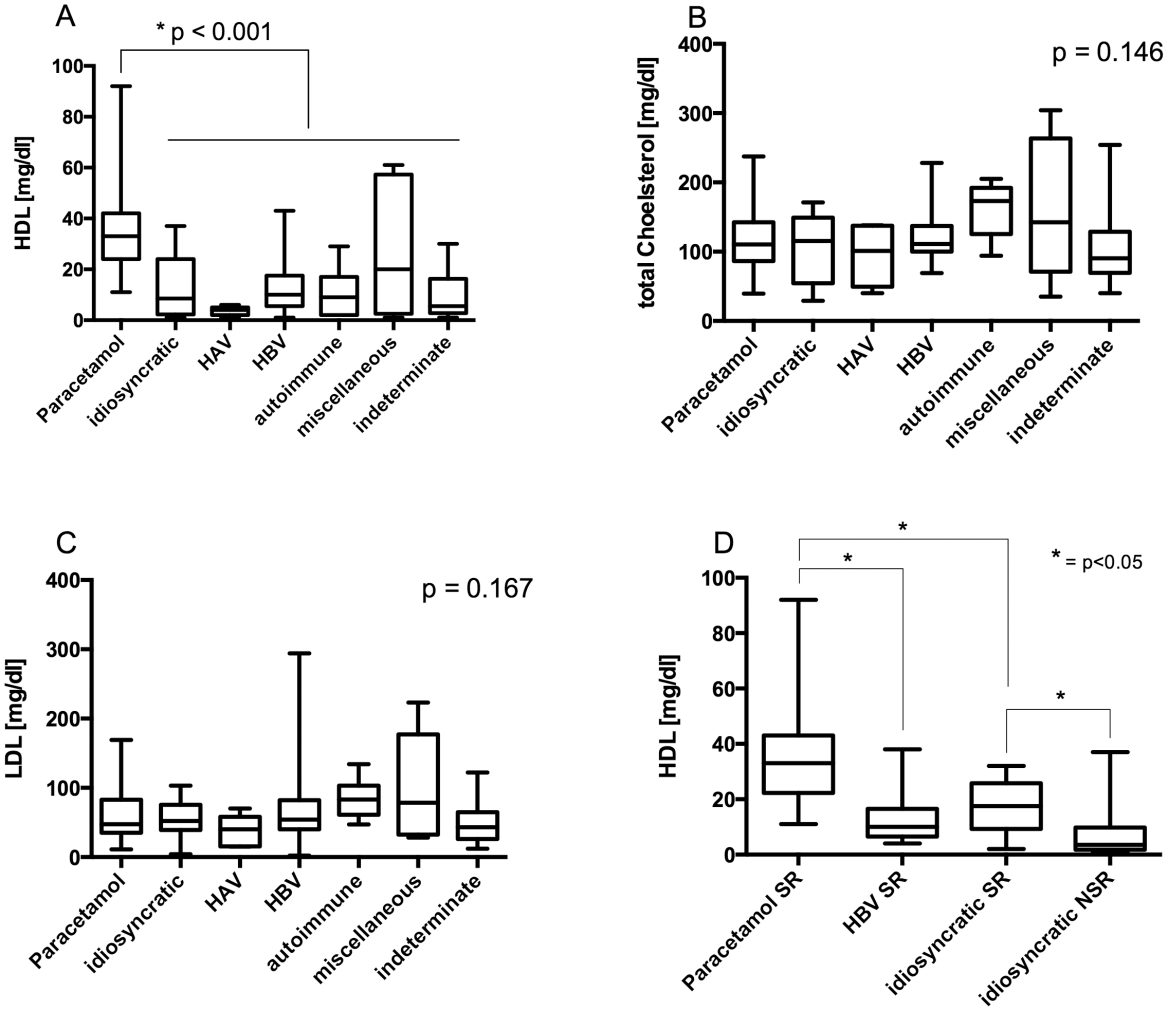
HDL levels differ between different etiologies. We compared the levels of the different lipids between the different etiological groups. (**A**) HDL levels are higher in patients in acute liver failure due to acetaminophen intoxication in comparison to all other compared etiologies. However no difference has been found for cholesterol or LDL (**B, C**). Comparison of the spontaneously recovered patient's data of the three biggest subgroups showed strong significant differences. Serum levels of HDL are also lower in non-spontaneous survivors within the group of DILI (**D**).

Distribution of the analyzed lipid parameters over the various etiologies was determined. Higher levels of HDL were found in acetaminophen induced liver failure compared to non-acetaminophen liver failure (fig. 3A). No significant differences in TC or TG were found between etiology groups (fig. 3B and 3C). However, serum levels of HDL have also been significantly lower in NSR subgroups of acetaminophen-induced ALF, HBV-induced ALF, and DILI (fig. 3D).

## Discussion

In the present study we confirm and expand current data on HDL and other lipid serum components in ALF. As previously shown by Atogo-Asse et al. [11], HDL and LDL were significantly reduced in ALF. Though, free cholesterol and TG were within common ranges. HDL positively correlated with classic liver parameters and liver function. Inverse correlations were found for HDL and the MELD score as well as INR and bilirubin, both indicators of diminished liver function. Moreover, HDL, cholesterol, and LDL were significantly lower in patients that required transplantation or those who deceased compared to SR patients. Since blood samples were taken at an early time point of ALF course, these lipid components might yield prognostic properties as demonstrated in ROC analyses. In addition, etiology specific distributions of HDL concentrations were observed and were predictive of outcome within specific etiology groups. Lipid serum components and in particular HDL might be good candidates for ALF outcome predictors.

During the last years reduced HDL was associated with aggravated disease course in cirrhosis and sepsis in critically ill patients [6]. As major cause for this observation reduced production of apolipoprotein IA was suggested, the most common protein associated to HDL particles. This would fit well with reduced liver function in chronic as well as acute liver diseases, since the liver is the main production site for apolipoproteins. In parallel no strong reduction in free cholesterol was observed in earlier studies [6], which is in line with the findings presented here. Apolipoprotein concentrations were unfortunately not available in the presented cohort. Thus, reduced HDL levels may be due to compromised liver function in cirrhosis and acute liver failure, which could indicate severity of the disease course and support the prognostic properties described by our findings.

Another important aspect of HDL is a function in liver regeneration. HDL delivers cholesterol and other lipid components to liver cells [2], where they are utilized as building blocks (i.e. for membranes) or metabolized to supply energy. Reduced HDL may be both: a sign and a cause for aggravated disease course in ALF. While the liver may contribute to reduced HDL availability due to impaired function, as described above, reduction of HDL may in turn decrease the capacity for regeneration within the liver. Liver regeneration requires a certain amount of fat supply, as we and others have shown [12], [13], [14]. Compromised energy and nutrient supply could thus be one cause for adverse outcome associated with diminished HDL serum concentrations.

In addition to the functions as energy supply and building block for membranes, HDL has been linked to innate immune processes. HDL can bind LPS of Gram-negative cell walls [7], [8]. In particular, LPS elicits a stronger response in monocytes of cirrhotic patients than in healthy monocytes *ex vivo*. This effect could be ameliorated by supplementing HDL [15]. In a murine model of sepsis, ApoA-I-knockout mice, with reduced HDL levels, were more susceptible to sepsis than wild type controls, while transgenic ApoA-I animals, with increased HDL levels, were moderately resistant to sepsis [16]. Finally in patients with sepsis low apolipoprotein A-I and HDL are associated with poor outcome [6]. The exact mechanism for the protective effects of HDL are not clear yet, but may be related to lipid droplets or microvesicles as carriers of danger-associated molecular patterns [17], [18]. Low HDL could aggravate detrimental effects of immune reactions and inflammatory process in ALF and would thus be indirectly associated to severity of the disease. By now pharmaceutical therapies for low HDL are available [19]. Though, prospective clinical trials would need to confirm, if these might support conservative treatment of ALF patients.

Taken together our data show that low HDL serum concentrations are associated with a negative outcome in ALF. While it is correlated to classic liver function parameters, it is not associated with cell death and could comprise an independent functional parameter with predictive properties for the disease course. More insight into the underlying mechanisms and connection to regenerative as well as inflammatory processes is needed to put our findings into a clinical perspective.
